# Mechanisms and therapeutic potential of mitochondrial-targeted therapies in bone repair

**DOI:** 10.1080/07853890.2026.2641277

**Published:** 2026-03-13

**Authors:** Nanjian Xu, Weihu Ma, Guanyi Liu, Fang Yang

**Affiliations:** a Spine Surgery Center, Ningbo No.6 Hospital, Ningbo, Zhejiang, China; b Ningbo Clinical Research Center for Orthopedics, Sports Medicine & Rehabilitation, Ningbo No.6 Hospital, Ningbo, Zhejiang, China; c Department of Nursing, Ningbo No.6 Hospital, Ningbo, Zhejiang, China

**Keywords:** Mitochondria, bone repair, energy metabolism, targeted therapy, reactive oxygen species, mitochondrial dynamics

## Abstract

Bone defect repair remains a significant challenge in orthopedics, particularly for critical-sized bone defects, which often result in nonunion. Traditional treatments have numerous limitations. Recent studies have highlighted the pivotal role of mitochondria as cellular energy and metabolic hubs influencing the function of osteoblasts, osteoclasts, and chondrocytes. Mitochondria regulate energy metabolism, ROS signaling, mitochondrial dynamics, and apoptosis, all of which are essential for maintaining proper bone function. Mitochondrial dysfunction has been identified as a key intrinsic factor contributing to the failure of bone repair. Thus, targeting mitochondria has emerged as a promising therapeutic strategy. This article systematically reviewed the various functional roles of mitochondria in bone repair and evaluated the current progress of mitochondrial-targeted therapeutic strategies. We focused on the mechanisms of action and preclinical advancements related to small molecule compounds, functionalized biomaterials, and advanced cell therapies, offering a theoretical foundation for their potential clinical application. Mitochondrial-targeted therapies show significant promise for enhancing bone repair by improving cellular energy metabolism, restoring redox homeostasis, optimizing mitochondrial quality control, and promoting cell survival. However, this field faces several challenges, including improving targeted delivery efficiency, ensuring long-term safety, and translating these strategies into clinical practice. Future research should prioritize the development of more precise delivery technologies, exploration of multi-target synergistic approaches, and rigorous clinical trials to support the practical application of mitochondrial-targeted therapies for clinical bone regeneration.

## Graphical Abstract

**Figure:**
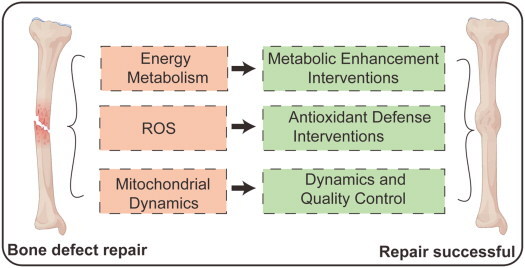

This review highlights the strong translational potential of mitochondrial-targeted therapies as a next-generation strategy for bone repair. By systematically linking specific mitochondrial dysfunctions to mechanism-based interventions. It provides a rational framework for developing treatments that enhance cellular fitness. Overcoming delivery and safety challenges through interdisciplinary efforts could revolutionize the management of critical-sized defects, particularly in compromised healing environments like aging or diabetes.

## 1. Introduction

Bone defects resulting from trauma, tumor resection, infection, and congenital deformities are common and present significant challenges in orthopedics. While bone has an impressive intrinsic repair capacity, healing fails when the defect exceeds a critical threshold, leading to nonunion or delayed union [1,2]. Nonunion occurs in approximately 5%-10% of all fractures, causing long-term pain, functional impairment, and a substantial decrease in quality of life for patients, along with a significant socioeconomic burden [3]. Critically, the underlying mitochondrial pathology often varies across different clinical etiologies. For instance, aged bone is frequently characterized by mitochondrial biogenesis decline and redox imbalance, diabetic bone by excessive glycolytic flux and mitochondrial oxidative stress, while inflammatory non-union may involve mitochondria-mediated NLRP3 inflammasome activation. Patients often require multiple complex surgical interventions, such as autologous or allogeneic bone grafting or distraction osteogenesis, which result in extended recovery periods, high medical costs, and lost productivity due to work absence [4]. Currently, autologous bone grafting is considered the ‘gold standard’ for bone repair due to its osteogenic, osteoconductive, and osteoinductive properties, without the risk of immune rejection. However, its application is severely limited by donor-site morbidity and limited bone mass [5,6].Allogeneic bone transplantation addresses the issue of bone mass but carries risks such as disease transmission, immune rejection, and poor integration [7,8]. These current challenges highlight a core issue: existing strategies primarily focus on providing the ‘raw materials’ (scaffolds) or ‘signaling instructions’ (growth factors) required for osteogenesis, while relatively neglecting the intrinsic factors that determine whether these ‘instructions’ can be effectively executed by cells, particularly the functional status of the cells themselves [9,10]. Consequently, the paradigm in bone repair is shifting from a traditional ‘material-centric’ perspective to a more fundamental ‘cell-centric’ one [11–17]. In this new paradigm, cellular function and health, particularly energy metabolism and survival status, are regarded as the ultimate bottleneck in determining the success or failure of bone repair. This urgently calls for a shift beyond traditional approaches and the exploration of innovative therapeutic strategies that directly enhance the function and vitality of osteoprogenitor cells, osteoblasts, and chondrocytes, ultimately overcoming current treatment limitations and providing safer, more effective, and reliable solutions for patients.

Mitochondria have traditionally been regarded as the ‘energy factories’ of eukaryotic cells, responsible for producing the majority of cellular ATP through oxidative phosphorylation and the tricarboxylic acid (TCA) cycle [18,19]. This function is particularly crucial for bone repair, as osteoblast differentiation, migration, and extracellular matrix production, are energy-intensive processes that require a continuous and sufficient ATP supply [20–22]. However, recent research over the past decade has radically reshaped this conventional view, revealing that mitochondria are not mere passive energy providers but dynamic, multifunctional centers of signal integration and regulation. These organelles actively control cell fate and tissue homeostasis, with profound implications for cellular and metabolic regulation [23,24]. Furthermore, mitochondria are the primary source of ROS, which have a complex, ‘double-edged sword’ role in cellular processes. Low physiological concentrations of ROS are crucial second messengers that positively regulate osteoblast differentiation, proliferation, and maturation through redox-sensitive signaling pathways, such as the activation of NF-κB and MAPK. However, under pathological conditions, dysfunction of complexes I and III in the mitochondrial electron transport chain leads to electron leakage, triggering excessive production of superoxide anions. These anions form hydrogen peroxide and hydroxyl radicals, resulting in persistent oxidative stress that damages mitochondrial DNA (mtDNA), lipids, and proteins. This oxidative damage eventually induces osteoblast and osteocyte apoptosis through pathways like JNK/p53 [25–27]. This redox imbalance is a common thread in many clinical bone repair failures, suggesting that antioxidant strategies like MitoQ might be broadly applicable, yet their efficacy may depend on the specific source of oxidative stress.Mitochondria are also central to cellular signaling by regulating Ca^2+^ homeostasis. As key intracellular calcium reservoirs, mitochondria take up Ca^2+^ *via* the mitochondrial calcium uniporter, which not only buffers cytosolic calcium levels but also directly regulates key enzymes in the TCA cycle. This coupling of electrical and metabolic signals affects processes ranging from metabolism to autophagy and apoptosis [28–31]. Moreover, mitochondria maintain their structural integrity and functional health through continuous fusion, fission, and selective autophagy, a process collectively known as mitochondrial dynamics [22,32]. Finally, mitochondria are central to the intrinsic apoptotic pathway. In response to apoptotic stimuli like DNA damage, endoplasmic reticulum stress, or growth factor deprivation, mitochondrial outer membrane permeability increases, allowing pro-apoptotic factors like cytochrome C and Smac/DIABLO to be released into the cytosol. These factors bind to Apaf-1, forming the apoptosome, which activates the caspase-9 and caspase-3 cascades, leading to programmed cell death [33,34]. This process is critical for both physiological bone remodeling and pathological bone loss. Mitochondria integrate energy metabolism, ROS signaling, calcium regulation, dynamics, and apoptosis into a complex and highly dynamic regulatory network. Therefore, the functional state of mitochondria directly determines whether repair cells have the necessary ‘capacity’ and ‘vitality’ to respond to osteogenic signals in the microenvironment, ultimately affecting the success of bone regeneration. Any factor that causes mitochondrial dysfunction can impair bone repair. Given the heterogeneity of mitochondrial dysfunction across clinical scenarios, a key future direction is to match specific interventions to patient stratification based on mitochondrial profiles. Aged bone with declined biogenesis might respond best to NAD+ boosters, whereas diabetic bone might require a combined approach targeting glycolytic reprogramming and antioxidant defense. Interventions aimed at improving mitochondrial health, therefore, represent promising strategies for promoting bone regeneration [35]. This comprehensive understanding of mitochondrial function lays a strong theoretical foundation for the development of precision therapeutic strategies targeting mitochondria.

**Figure 1.**
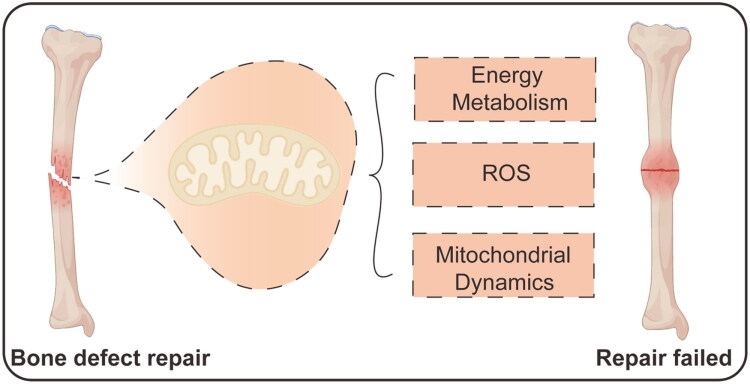
Mitochondria: key player in bone repair outcomes.

Accordingly, this review is structured to systematically explore the potential of targeting mitochondrial function as an innovative strategy for enhancing bone regeneration. It is organized around a clear ‘problem-to-solution’ logic flow: beginning with the clinical limitations of current bone repair strategies, it establishes mitochondria as a central regulatory node and a modifiable target. The core of the article presents a mechanism-based classification of mitochondrial dysfunction in bone repair—categorizing key pathological imbalances in metabolism, redox homeostasis, dynamics, and quality control—and meticulously aligns each imbalance with its corresponding category of intervention strategies. The discussion encompasses the mechanisms of action, preclinical evidence, and translational potential of these strategies. Finally, the review objectively addresses prevailing challenges and outlines a forward-looking, interdisciplinary trajectory for future progress, encompassing advanced targeting technologies, multi-target synergistic approaches, and a defined clinical translation pathway, aiming to provide a comprehensive and insightful resource for advancing mitochondrial-targeted bone repair therapies (Figure 1).

This illustration contrasts successful bone defect repair with failed repair, highlighting how mitochondrial functions like energy metabolism, ROS signaling, and dynamics critically influence the healing process.

## 2. Mitochondrial function during bone repair

### 2.1. Energy metabolism and biosynthesis

**Figure 2.**
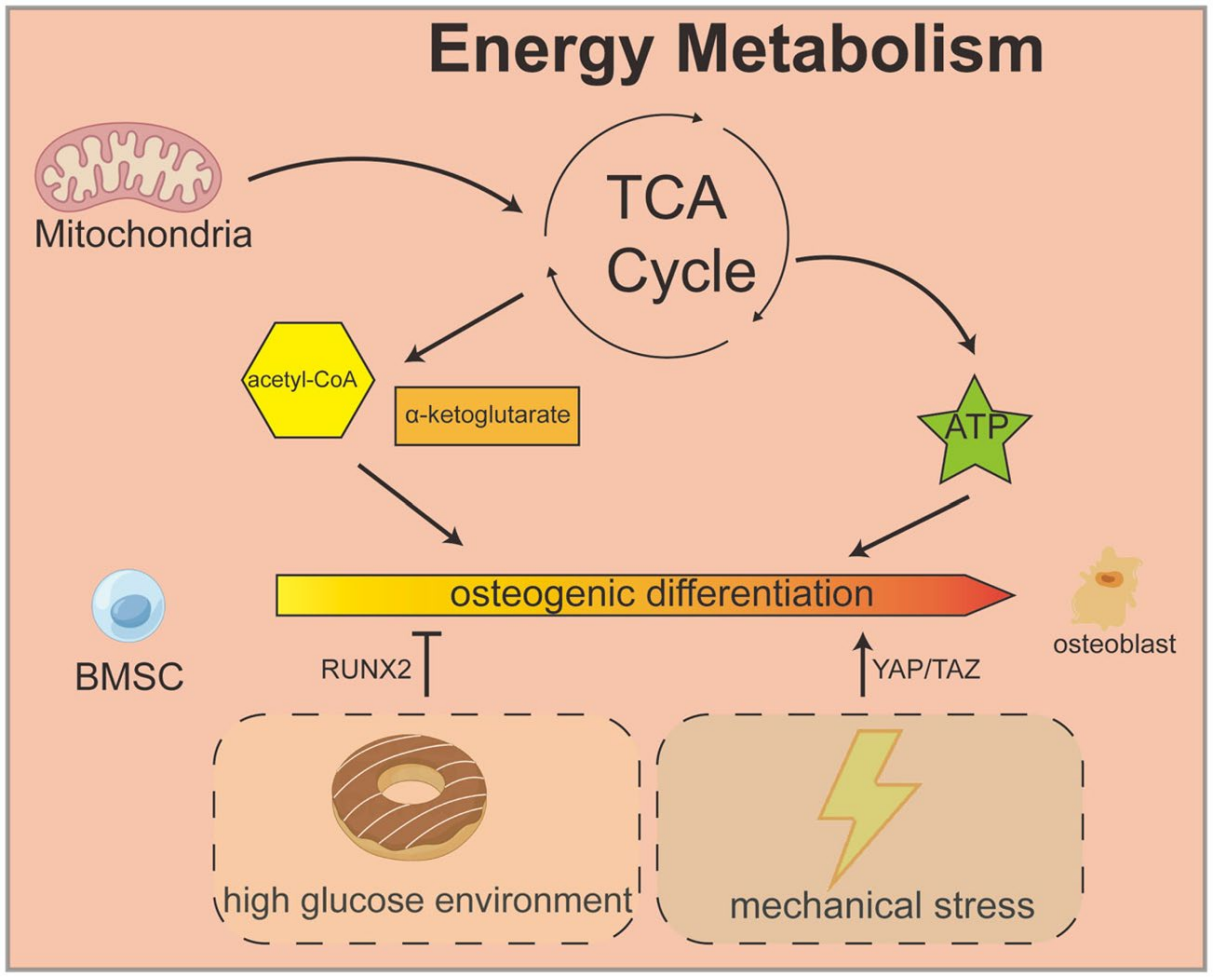
Energy metabolism and biosynthesis during bone repair.

During bone repair, osteoblast differentiation and mineralization are highly energy-demanding processes, critically dependent on efficient energy metabolism and biosynthesis provided by mitochondria. The differentiation of mesenchymal stem cells (MSCs) into osteoblasts involves not just morphological changes but profound biosynthetic remodeling. This includes the transcription and translation of numerous genes, the synthesis and secretion of extracellular matrix proteins, and the biomineralization of hydroxyapatite crystals. The mineralization process, which pumps inorganic mineral ions out of the cell to form crystals, heavily relies on the energy generated by ATP hydrolysis. Therefore, cells must significantly boost their energy production capacity to meet these demands. Mitochondria, often referred to as the ‘energy factories’ of the cell, generate substantial amounts of ATP through OXPHOS, providing the energy required for osteoblasts to synthesize key proteins such as collagen and alkaline phosphatase and to deposit calcium and phosphate crystals. Studies have shown that during osteoblast differentiation from human MSCs, mitochondrial activity is significantly enhanced, evidenced by increases in oxygen consumption rate and maximal respiratory capacity. This suggests that upregulation of OXPHOS activity forms the metabolic foundation for osteogenic differentiation [36]. Furthermore, intermediates of mitochondrial metabolism play roles beyond just energy production; they actively participate in regulating gene expression that influences cell fate. Metabolites such as acetyl-CoA and α-ketoglutarate, produced in the TCA cycle, serve as key substrates or cofactors for epigenetic modifications like histone acetylation and methylation. These modifications regulate the chromatin accessibility of osteoblast-related genes, precisely steering the osteogenic differentiation program. A key molecule that maintains this efficient metabolic state is nicotinamide adenine dinucleotide (NAD+), an essential coenzyme in the electron transport chain whose levels directly influence the efficiency of OXPHOS. In young, non-aging MSCs, a high-glucose environment depletes NAD+ by promoting glycolysis, leading to a decrease in the NAD+/NADH ratio. This imbalance impairs mitochondrial membrane potential and OXPHOS function, ultimately pushing the cell fate toward adipogenic differentiation instead of osteogenic differentiation [37]. This metabolic reprogramming, coupled with changes in the epigenetic landscape, illustrates the interconnected regulation of energy metabolism and gene expression during bone formation. Moreover, under mechanical stress, such as the cyclic tensile force during orthodontic tooth movement, bone marrow stromal cells experience a moderate increase in ROS production, accompanied by a compensatory increase in NAD+ levels. This boost activates antioxidant pathways, including NRF2, which helps maintain redox homeostasis and ensures proper osteogenic differentiation [38]. Therefore, targeted interventions in NAD+ biosynthesis or supplementation with its precursors have emerged as potential therapeutic strategies to promote bone repair and accelerate bone regeneration by enhancing mitochondrial function (Figure 2).

Mitochondria are involved in the core mechanisms of energy metabolism and biosynthesis during bone repair. Under mechanical stress or high-glucose conditions, BMSC mitochondria accelerate metabolism through the TCA cycle, producing large amounts of ATP and acetyl-CoA. The latter not only provides energy for cells but also acts as a key signaling molecule to directly promote chromatin remodeling and histone acetylation, thereby driving the expression of osteogenic genes, ultimately guiding BMSC differentiation into osteoblasts and completing bone tissue regeneration.

### 2.2. Signaling role of ROS

**Figure 3.**
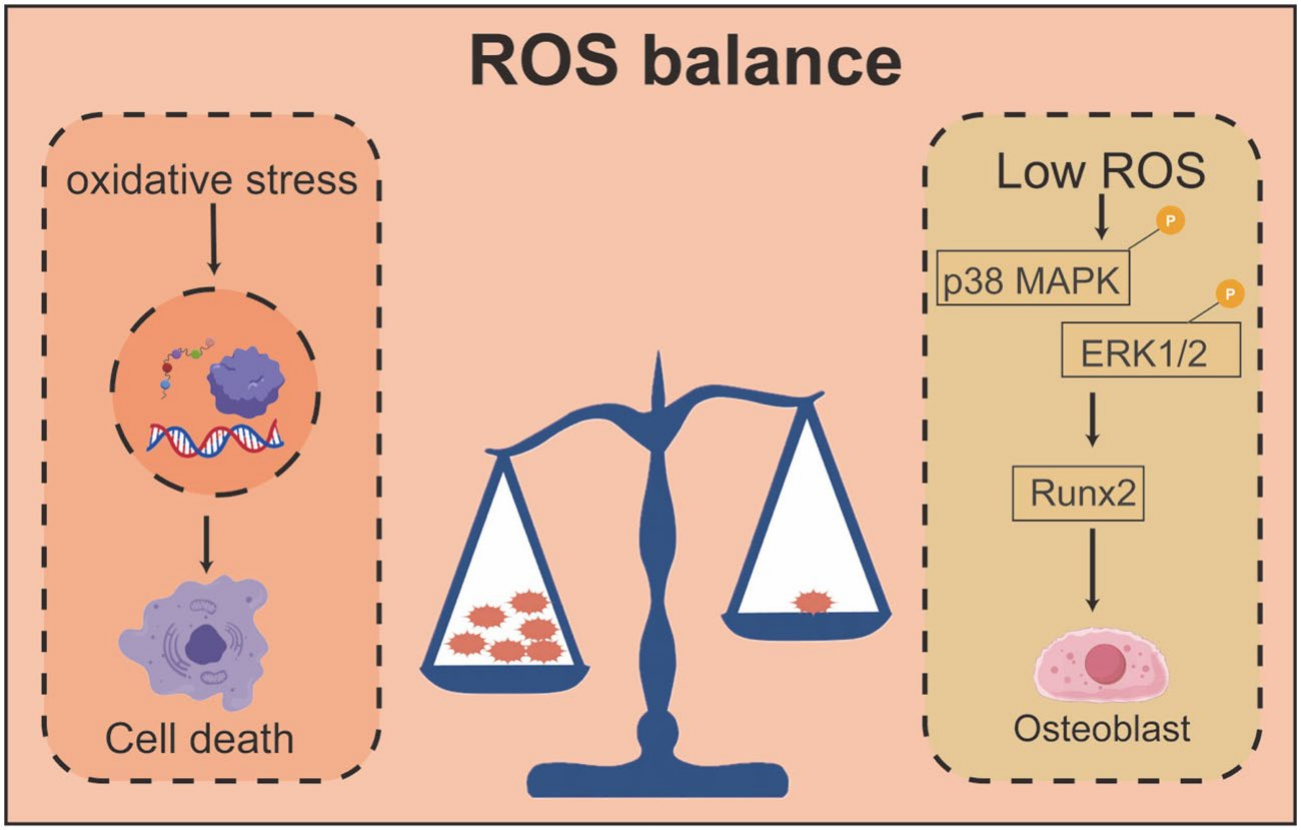
Signaling role of ROS during bone repair.

ROS play a complex and dual role in bone repair, with their biological effects heavily dependent on their intracellular concentration, duration, and site of production. While ROS are often perceived as harmful byproducts, they are actually critical signaling molecules that influence osteoblast fate and determine the success or failure of bone repair. Under physiological conditions, low to moderate levels of ROS are not detrimental but serve as important second messengers that positively regulate osteoblast differentiation *via* redox signaling pathways. The mitochondrial electron transport chain and the NADPH oxidase family are the primary sources of intracellular ROS [39]. In response to osteogenic signals such as BMPs and Wnt, cells generate moderate amounts of ROS, which oxidatively modify cysteine residues in specific signaling proteins, thereby activating downstream cascades. For example, low concentrations of hydrogen peroxide (H_2_O_2_) enhance the phosphorylation of p38 MAPK and ERK1/2, upregulating the expression and activity of the core transcription factor Runx2. This promotes alkaline phosphatase activity and extracellular matrix mineralization, driving the differentiation of MSCs into osteoblasts [39]. Importantly, ROS signaling is tightly regulated, with ROS generation balanced by the intracellular antioxidant system to maintain ‘redox homeostasis’, which supports the bone formation microenvironment [39]. However, when ROS production surpasses the capacity of antioxidants to neutralize them, ROS shift from beneficial signaling molecules to damaging mediators, triggering oxidative stress. In pathological conditions such as high glucose, aging, inflammation, or ischemia, mitochondrial dysfunction leads to electron leakage from the electron transport chain, resulting in excessive production of superoxide anions (O_2_•^-^) [39]. These excess ROS irreversibly damage lipids, proteins, and DNA, initiating a cascade of harmful effects: collapse of mitochondrial membrane potential, failure of ATP synthesis, release of proinflammatory cytokines, and, ultimately, osteoblast and osteocyte apoptosis *via* the mitochondrial pathway [40]. More critically, sustained high ROS levels not only directly damage osteoblasts but also abnormally activate osteoclast differentiation. The RANKL signaling pathway, which is crucial for osteoclast activation, relies on ROS as a second messenger. Excessive ROS exacerbate NFATc1 activation, leading to increased bone resorption and disrupting the delicate balance between bone formation and resorption. This imbalance is a key pathological driver in conditions like osteoporosis and nonunion [41]. Therefore, targeted regulation of ROS levels has emerged as a promising strategy for enhancing bone repair. The objective is not to eliminate ROS entirely but to precisely control their levels within the physiological range that supports osteogenic signaling. This can be achieved through the use of smart antioxidants or by activating endogenous antioxidant defense systems, which would promote bone regeneration while inhibiting pathological bone loss [42] (Figure 3).

This diagram reveals the bidirectional signaling role of ROS in bone repair. It visually demonstrates the importance of ROS balance: high ROS (oxidative stress) on the left leads to DNA damage and cell death; low ROS on the right activates key signaling pathways such as p38 MAPK and ERK1/2, upregulating the expression of the key osteogenesis transcription factor Runx2, ultimately promoting osteoblast differentiation and achieving bone regeneration. This diagram emphasizes the critical role of maintaining precise ROS levels in successful bone repair.

### 2.3. Mitochondrial dynamics and quality control

**Figure 4.**
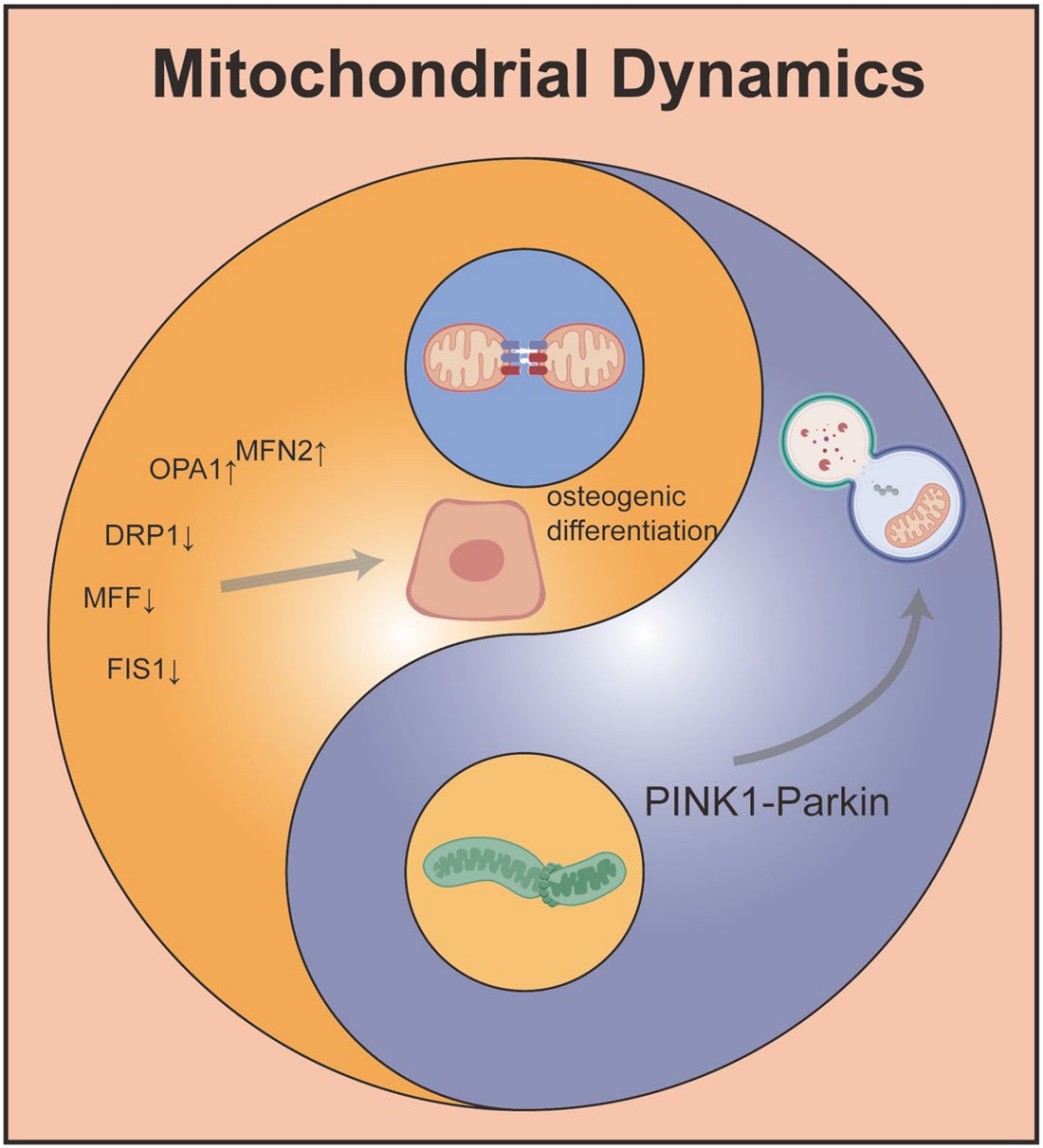
Role of mitochondrial dynamics in bone repair.

Mitochondrial dynamics and mitophagy are essential mechanisms for maintaining mitochondrial quality control and play crucial roles in bone metabolism and regeneration [43,44]. Mitochondrial dynamics preserve the morphological and functional integrity of the mitochondrial network through continuous fusion and fission processes. Fusion, mediated by mitofusin 1 (MFN1), MFN2, and optic atrophy protein 1 (OPA1), facilitates the exchange of mitochondrial contents and helps dilute damaged components. Fission, mediated by dynamin-related protein 1 (DRP1) and its receptor, mitochondrial fission factor (MFF), isolates damaged components for clearance through autophagy [45,46]. During bone formation, osteoblast differentiation is associated with a shift in energy metabolism from glycolysis to oxidative phosphorylation, requiring mitochondria to possess efficient energy production capacity. Studies have shown that graphene oxide quantum dots (GOQDs) can enhance the osteogenic differentiation of human periodontal ligament stem cells (hPDLSCs) by promoting mitochondrial fusion and inhibiting excessive fission. This is reflected in the upregulation of MFN2 and OPA1 expression, along with decreased expression of DRP1, MFF, and FIS1, thereby maintaining mitochondrial network elongation and supporting osteogenic differentiation [45]. On the other hand, an imbalance in mitochondrial dynamics can lead to functional abnormalities. For example, under conditions of inflammation or oxidative stress, excessive mitochondrial fission leads to fragmentation, elevated ROS production, and decreased ATP synthesis, ultimately impairing osteogenic function [47]. Mitochondrial autophagy, which involves the selective clearance of damaged mitochondria, is crucial for maintaining cellular homeostasis. The PINK1-Parkin pathway is a classical mitophagy pathway: when the mitochondrial membrane potential decreases, PINK1 stabilizes on the outer mitochondrial membrane and recruits Parkin, initiating ubiquitination. PINK1 then binds to LC3 through adaptor proteins such as p62, forming autophagosomes that fuse with lysosomes for degradation [48]. Studies have shown that downregulation of PINK1 expression in osteoporosis patients and animal models impairs mitochondrial autophagy and reduces osteogenic differentiation [49]. Activating mitochondrial autophagy can promote mitochondrial calcium release and amorphous calcium phosphate secretion, accelerating matrix mineralization [48]. Additionally, the deacetylase Sirtuin 1 (SIRT1), which alleviates mitochondrial oxidative stress by activating superoxide dismutase 2 (SOD2), is a key regulator of autophagy. Melatonin has been shown to restore mitochondrial function in BMSCs from osteoporotic rats through the SIRT1-SOD2 axis and promote osteogenesis [50]. There is also significant cross-talk between mitochondrial dynamics and autophagy. For example, fragmented mitochondria produced by mitochondrial fission are more readily cleared by autophagy, while the fusion process helps prevent unnecessary activation of autophagy [51]. During aging, histone deacetylase 2 (HDAC2)-mediated deacetylation of Parkin inhibits mitochondrial autophagy, leading to a decline in the osteogenic capacity of hPDLSCs. Restoring Parkin acetylation levels can enhance autophagic flux and reverse age-related declines in osteoblast function [51]. Similarly, copper ions can activate PINK1/Parkin-mediated autophagy and promote osteoblast mineralization by inducing mitochondrial fission and increasing mitochondrial ROS production [48]. In summary, mitochondrial dynamics and mitophagy work together to maintain bone cell homeostasis and function by regulating energy metabolism, oxidative stress, and intracellular signaling. Targeting these mechanisms may offer novel therapeutic strategies for osteoporosis and bone defect repair (Figure 4).

This picture uses the Tai Chi diagram to vividly illustrate the balancing role of mitochondrial dynamics in bone repair: mitochondrial fusion on the left promotes osteogenic differentiation, and the PINK1-Parkin pathway on the right mediates mitochondrial autophagy, jointly regulating the repair process.

## 3. Therapeutic strategies targeting mitochondria

Mitochondrial dysfunction represents a cornerstone pathology in compromised bone repair, manifesting as a complex interplay of bioenergetic deficits, redox imbalance, structural disintegration, and quality control failure. By systematically addressing the core pillars of mitochondrial dysfunction—impaired metabolism, oxidative stress, dynamical instability, and irreparable damage—we can construct a comprehensive arsenal of strategies for bone regeneration. This section delineates these mechanism-based interventions into four coherent categories: metabolic enhancement, antioxidant defense and redox homeostasis, dynamics and quality control remodeling, and mitochondrial replacement and functional reconstitution, each supported by evidence from bone-related studies and analogous pathological models. In addition, Table 1 summarizes the small molecule compounds targeting mitochondria.

**Table 1.** Summary of research on small molecule compounds targeting mitochondria.

Category	Representative compounds	Mechanism	Key research models and findings	References
Mitochondrial-targeted antioxidants	MitoQ	By targeting mitochondria, TPP+ eliminates ROS, reduces oxidative damage (MDA, H_2_O_2_), and improves mitochondrial membrane potential and cell viability.	Bull semen freezing: 50–500 nM improves sperm motility and membrane integrity	[60]
			Cryopreservation of yak semen: 200 nM enhances T-AOC and SOD activities	[61]
	MT	Nitrogen oxides target mitochondria, inhibit ROS generation, and restore antioxidant enzyme activity.	MT is more effective than SkQ1 in alleviating oxidative damage in renal ischemia-reperfusion	[62]
Energy metabolism regulators	NMN	Increase NAD+ levels, activate SIRT1, enhance mitochondrial UPRmt and autophagy, and improve energy metabolism.	AD model: NMN improves mitochondrial stress through the ATF4-UPRmt pathway	[52]
			PD model: enhancing MQC and reducing inflammation	[54]
	NAD+/O₂ synergistic delivery system	MnO₂ nanozymes catalyze H₂O₂ to produce O₂, and combine with NAD+ precursors to restore the function of the respiratory chain.	Osteoarthritis: Hydrogel delivery system improves chondrocyte mitochondrial function	[57]
Mitochondrial dynamics regulators	LCN2 inhibitors	Blocking LCN2-mediated OMA1 degradation and OPA1 accumulation restores mitochondrial fusion/fission balance	Periodontitis: Macrophage Mito-EVs disrupt BMSCs mitochondrial morphology *via* LCN2	[63]
	MFN1/2 or OPA1 activators	Promotes mitochondrial fusion, maintains network stability, and supports high energy demand processes	Mitochondrial fusion defects are associated with functional decline of BMSCs	[63]

MT: MitoTEMPO; NMN: NAD+ precursor; MDA: malondialdehyde; T-AOC: total antioxidant capacity; SOD: superoxide dismutase; UPRmt: unfolded protein response in mitochondria; LCN2: lipocalin-2; OMA1: optic atrophy 1 metalloprotease; OPA1: optic atrophy protein 1; MFN: mitofusin; AD: Alzheimer’s disease; PD: Parkinson’s disease; MQC: mitochondrial quality control; Mito-EV: mitochondria-enrich extracellular vesicles; BMSC: bone marrow stromal cell.

### 3.1. Metabolic enhancement interventions

**Figure 5.**
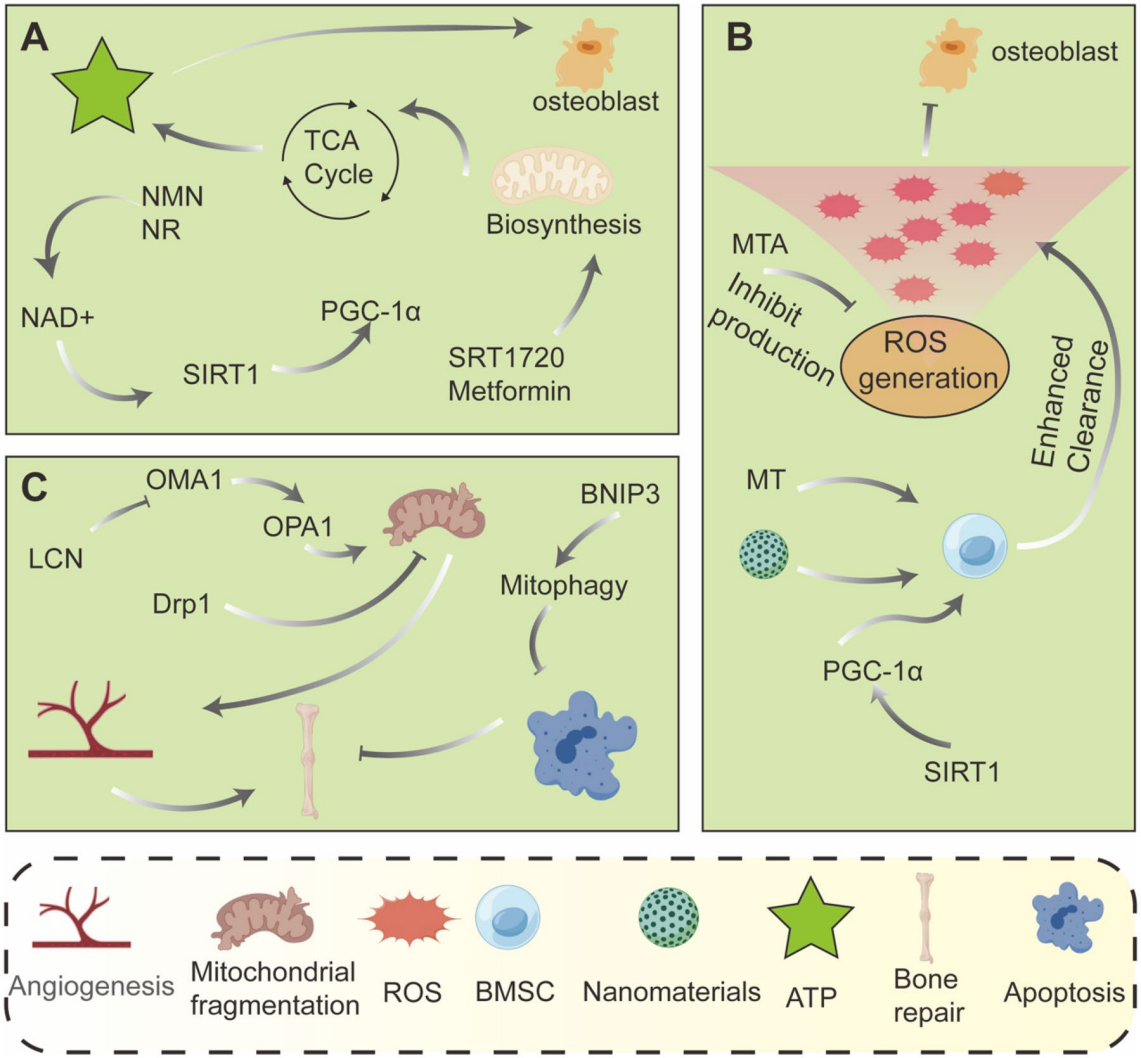
Mitochondrial mechanisms in bone regeneration. (A) NAD+ precursors boost ATP production *via* the TCA cycle and SIRT1-PGC-1α pathway to energize osteoblasts.(B) Nanomaterials scavenge excess ROS and activate antioxidant defenses through the SIRT1-PGC-1α axis, protecting cells. (C) Key regulators like Drp1 and OPA1 control mitochondrial fission/fusion, while BNIP3 mediates mitophagy to maintain healthy networks.

The osteogenic lineage commitment and matrix mineralization executed by bone-forming cells are extraordinarily energy-demanding processes, placing an immense burden on mitochondrial OXPHOS for ATP provision. A critical mechanism underlying failed bone repair, particularly in aging, diabetes, and inflammatory conditions, is the collapse of cellular energy metabolism. This collapse stems from diminished substrate availability, impaired ETC function, and dysregulated metabolic signaling, ultimately creating an energetic deficit that is incompatible with successful regeneration. Therapeutic strategies aimed at reinvigorating mitochondrial metabolism therefore seek to replenish metabolic cofactors, supply energy substrates, and amplify metabolic signaling pathways (Figure 5A).

#### 3.1.1. NAD^+^ boosting strategies

The decline in NAD^+^ levels is a fundamental hallmark of aging and metabolic syndromes, directly contributing to mitochondrial inefficiency. NAD^+^ serves as an essential co-substrate for sirtuins, a class of deacetylases critical for metabolic homeostasis. SIRT1, predominantly nuclear, deacetylates and activates PGC-1α, the master regulator of mitochondrial biogenesis. Concurrently, the mitochondrial sirtuin SIRT3 deacetylates and modulates the activity of numerous enzymes involved in the TCA cycle, fatty acid oxidation, and the ETC, thereby optimizing metabolic function. Crucially, these functional enhancements are underpinned by improvements in mitochondrial ultrastructure, such as increased cristae density and membrane integrity, which are essential for efficient electron transport and ATP synthesis. Supplementation with NAD^+^ precursors like nicotinamide mononucleotide (NMN) or nicotinamide riboside (NR) has demonstrated efficacy in restoring NAD^+^ pools across diverse models. In neuronal studies, NMN administration rectified the NAD^+^ metabolic profile, bolstered the mitochondrial unfolded protein response (UPRmt)—a key stress response pathway—and ameliorated pathology in models of Alzheimer’s and Parkinson’s diseases [52–54]. The relevance to skeletal tissue is profound. The osteogenic potential of BMSCs markedly diminishes with age, a phenomenon closely linked to NAD^+^ depletion and reduced SIRT3 activity [55]. More acutely, hyperglycemic conditions can precipitate a rapid NAD^+^ deficit in MSCs within hours, skewing their differentiation away from osteogenesis and towards adipogenesis *via* the NAD^+^/SIRT1 axis. Crucially, oral NAD^+^ precursor supplementation can swiftly reverse this metabolic dysfunction and restore lineage fidelity within days [56]. This compelling evidence positions NAD^+^ augmentation as a primary strategy for counteracting age-related and metabolic bone loss.

#### 3.1.2. Eenergy substrate supply and respiratory chain reactivation

Beyond cofactor replenishment, directly ‘feeding’ the ETC or repairing its components represents a direct metabolic rescue strategy. A groundbreaking example comes from osteoarthritis research, where chondrocytes exhibit ETC dysfunction. Researchers engineered a charge-guided micro-nanointerpenetrating network hydrogel for the simultaneous delivery of an NAD^+^ precursor (electron donor) and a MnO_2_-based nanozyme (electron acceptor). This innovative system effectively bypasses defective ETC complexes by providing both the initial electron donor (fuel) and the terminal electron acceptor, thereby reactivating the entire respiratory chain, alleviating cellular senescence, and preserving the extracellular matrix [57]. This metabolic reactivation is directly linked to the stabilization of ETC supercomplex assembly and the restoration of normal cristae architecture. This concept of ‘metabolic bypass’ is highly applicable to bone repair sites, which are often characterized by hypoxia and inadequate nutrient supply, leading to metabolic paralysis. Providing alternative energy substrates or employing nanozymes to mimic ETC function could potentially resurrect energy production in the distressed bone microenvironment.

#### 3.1.3. SIRT1 activators and AMPK signaling

Direct pharmacological activation of key metabolic sensors offers an alternative to substrate supplementation. SIRT1 activators (SRT1720) and AMPK activators (Metformin) can directly stimulate PGC-1α activity and mitochondrial biogenesis, independent of NAD^+^ levels. The efficacy of modulating this axis is supported by biomaterial studies. For instance, an oral delivery system based on fisetin-loaded liposomes within hydrogel microspheres exerted its osteoprotective effect in osteoporosis by activating the AMPK-SIRT1 pathway, thereby reversing mitochondrial senescence in BMSCs [58]. Similarly, the beneficial effects of a nanocomposite hydrogel containing sulfur quantum dots were partly attributed to improved ATP synthesis and potential modulation of the NAD^+^/SIRT1 axis, driving an anti-inflammatory macrophage phenotype conducive to healing [59]. These functional benefits are often consequent to the restoration of a healthy mitochondrial network morphology and the optimization of inner membrane systems for energy transduction. These findings validate the targeting of SIRT1 and AMPK as a potent metabolic enhancement strategy.

### 3.2. Antioxidant defense and redox homeostasis interventions

The inflammatory phase of bone repair, while necessary, generates a burst of ROS that can overwhelm endogenous antioxidant defenses, leading to oxidative stress. Mitochondria are both a primary source and a key target of excessive ROS. Oxidative damage to mitochondrial DNA, lipids, and proteins impairs ETC function, perpetuates ROS production, and triggers apoptotic pathways, ultimately compromising the survival and function of osteoprogenitor cells [64,65]. Strategies focused on restoring redox balance aim to precisely neutralize mitochondrial ROS without disrupting essential redox signaling (Figure 5B).

#### 3.2.1. Mitochondria-targeted antioxidants (MTAs)

MTAs represent a pinnacle of precision medicine in antioxidant therapy [66–69]. By conjugating an antioxidant moiety (e.g. ubiquinone, tocopherol) to a lipophilic cation like triphenylphosphonium (TPP^+^), these compounds accumulate 100–1000 fold within the mitochondrial matrix, driven by the large inner membrane potential. MitoQ, the most extensively studied MTA, has demonstrated robust cytoprotective effects across myriad models, from improving sperm quality during cryopreservation by reducing oxidative damage to protecting against ischemia-reperfusion injury [60,61]. The critical importance of targeted delivery is highlighted by comparative studies: while non-targeted antioxidants like SOD or coenzyme Q10 (CoQ10) show limited efficacy, MTAs like MitoQ and MitoTEMPO (MT) provide superior protection at significantly lower concentrations by acting precisely at the site of ROS generation [70]. Furthermore, the choice of MTA is crucial, as their pharmacological profiles differ; for instance, MT offered better renal protection than SkQ1 in one model, and high doses of SkQ1 proved cytotoxic [62,71,72], underscoring the need for careful agent selection and dosing.

#### 3.2.2. Smart antioxidant biomaterials

To overcome the transient action of small molecules and provide sustained, context-dependent antioxidant therapy, advanced biomaterials have been engineered [73]. These ‘smart’ systems can respond to the specific pathological microenvironment. A prime example is the ‘chameleon-like’ nanoplatform (HA@Ce-TA) for infected bone defects. Its design allows it to exhibit pro-oxidant properties (ROS generation for bacteria killing *via* photothermal therapy) in the acidic extracellular environment, but switch to potent antioxidant and pro-fusion activities once internalized by host cells, thereby comprehensively rescuing mitochondrial homeostasis [74]. Another sophisticated system is a cerium-based nanosystem (CNS) that combines ROS-scavenging capabilities with autophagy induction. In aged bone, this CNS activated the SIRT1-PGC-1α axis, promoted mitochondrial biogenesis and transfer, and functionally rejuvenated BMSCs [75]. These materials represent a significant advancement, providing spatially and temporally controlled redox regulation that is adaptable to the dynamic bone healing process.

### 3.3. Dynamics and quality control remodeling

Mitochondria are not static organelles but exist in a highly dynamic equilibrium, continuously undergoing cycles of fusion (interconnection) and fission (division) [76]. This dynamism is the cornerstone of mitochondrial quality control. The process of fusion allows for the mixing of contents between different mitochondria, thereby diluting localized damage (such as mutated mtDNA or oxidatively damaged proteins) and enabling functional complementation [77]. Conversely, fission enables the cell to isolate damaged mitochondrial segments, which are then cleared *via* a selective form of autophagy known as mitophagy. This ‘fusion-fission-mitophagy’ cycle is the core mechanism for maintaining the health of the mitochondrial network [78]. However, under pathological conditions, this delicate balance can be disrupted. An imbalance favoring excessive fission or impaired fusion leads to fragmentation of the mitochondrial network, resulting in bioenergetic failure, increased ROS production, and ultimately the triggering of apoptosis. Conversely, uncontrolled fusion can allow damaged components to spread throughout the network, amplifying their toxic effects [79]. Therefore, therapeutic interventions aim to restore this delicate kinetic balance and enhance overall quality control mechanisms through pharmacological or biomaterial approaches, which is crucial for maintaining the function of osteoblasts and their progenitors during bone repair (Figure 5C).

#### 3.3.1. Regulating the fusion/fission balance

Pathological disruption of mitochondrial dynamics is a key mechanism in various skeletal diseases. Understanding the specific manifestations of these alterations and their molecular basis is a prerequisite for developing targeted therapeutic strategies. A seminal study on periodontitis provided profound insight into this aspect. It elucidated that macrophage-derived extracellular vesicles (Mito-EVs) could deliver both damaged mitochondria and high levels of LCN2 to BMSCs. LCN2 played a critical pathogenic role: it induced the degradation of the mitochondrial peptidase OMA1, which is essential for regulating the key fusion protein OPA1. Dysregulation of OMA1 subsequently led to loss of OPA1 function, causing mitochondrial network fragmentation and ultimately impairing the osteogenic differentiation capacity of BMSCs. Importantly, inhibiting LCN2 rescued this mitochondrial defect and osteogenic dysfunction [63]. This finding highlights the therapeutic potential of targeting specific nodes within the dynamics regulatory pathway. Based on these mechanistic insights, researchers are actively exploring various strategies to modulate the dynamics balance. On one hand, strategies to promote mitochondrial fusion can be developed, such as using agonists for MFN1, MFN2, or OPA1, to help restore network connectivity and facilitate content sharing and functional complementation. On the other hand, inhibiting excessive fission is equally important, for instance, by applying Drp1 inhibitors (like Mdivi-1) to prevent excessive mitochondrial fragmentation [80,81]. Evidence supporting this regulatory approach comes from multiple areas. Research on diabetic wound healing found that silicon could modulate mitochondrial fission in macrophages *via* the Drp1-Mff signaling pathway; this modulation facilitated the transfer of functional mitochondria from macrophages to endothelial cells, thereby accelerating vascularization and the healing process [82]. This indicates that regulating dynamics is an effective means of improving intercellular communication and tissue regeneration. Furthermore, mechanical stimulation has also been proven to be an important regulator of dynamics. Studies have found that applying mechanical stretch to macrophages triggers enhanced Drp1-mediated mitochondrial fission, and subsequently, these macrophages show increased mitochondrial donation to BMSCs [83]. This reveals the intrinsic link between the mechanical environment, mitochondrial dynamics, and intercellular energy support, offering a new perspective for guiding bone regeneration using the mechanical properties of biomaterials.

#### 3.3.2. Enhancing mitophagy and biogenesis

When mitochondrial damage is confined to specific segments, their timely removal *via* mitophagy is crucial. Mitophagy is a multi-step process involving the recognition of damaged mitochondria, their encapsulation by autophagosomes, and degradation by fusion with lysosomes [84]. Enhancing this quality control pathway can effectively ‘clean out’ dysfunctional organelles, making space for new, healthy mitochondria [85]. Biomaterial engineering provides innovative solutions for precisely activating mitophagy [86]. A prime example is a biomimetic nanocoating constructed on the surface of a magnesium alloy. This coating not only modulates the degradation of the magnesium alloy to match the bone healing timeline but, more importantly, specifically upregulates the expression of the mitophagy receptor BNIP3. The upregulation of BNIP3 guides the cells to precisely clear dysfunctional mitochondria, thereby reducing osteoblast apoptosis and ultimately significantly accelerating bone regeneration in an osteoporotic rat model [87]. This strategy of targeted activation of a specific autophagy receptor represents a precise quality control method, avoiding potential side effects of non-selective autophagy activation. However, merely clearing damaged units is insufficient; replenishing new, fully functional mitochondria is equally critical, which involves mitochondrial biogenesis—the generation of new mitochondria. Promoting biogenesis can fundamentally renew and expand the pool of healthy mitochondria, providing cells with greater metabolic capacity. As mentioned earlier, various interventions can effectively enhance mitochondrial biogenesis. CNS was able to activate the SIRT1-PGC-1α axis *in situ* within the aged bone microenvironment; this pathway is a primary driver of mitochondrial biogenesis, thereby promoting the generation of new mitochondria in BMSCs [75]. Similarly, supplementing with NAD^+^ precursors (like NMN) activates SIRT1 by elevating NAD^+^ levels, which in turn can enhance PGC-1α activity [88]. The promotion of biogenesis and improved mitochondrial function have been observed in various disease models with impaired mitochondrial function [52]. Therefore, an ideal therapeutic strategy often requires synergistic action: on one hand, enhancing mitophagy to ‘remove the undesirable’, and on the other, promoting biogenesis to ‘supply the new’. Only by addressing both aspects can the functional remodeling of the mitochondrial network be achieved most effectively, providing a solid energy foundation for successful bone repair.

### 3.4. Mitochondrial replacement and functional reconstitution

In the context of bone repair, numerous pathological conditions—such as advanced age, prolonged metabolic stress, or severe inflammation—can inflict damage upon mitochondria that is too extensive for endogenous repair mechanisms or pharmacological modulation to correct. When mitochondrial dysfunction reaches a point of severity where the integrity of the organelle’s structure, its DNA, or its core enzymatic machinery is irreparably compromised, simply attempting to enhance its existing function becomes insufficient [89]. In these cases of severe or irreversible mitochondrial damage, the most radical yet logically definitive therapeutic strategy is to directly replace the defective organelles or their essential molecular components. This approach represents a fundamental shift from merely modulating the activity of ailing mitochondria to executing a true ‘hardware’ replacement, offering a potent solution for cellular energy rescue by introducing fully functional units. The strategies under this paradigm can be broadly implemented through several innovative modalities, each with distinct mechanisms and applications.

#### 3.4.1. Cell-mediated mitochondrial transfer

This strategy ingeniously harnesses and amplifies a natural physiological process known as intercellular mitochondrial transfer, which is increasingly recognized as a fundamental mechanism for tissue homeostasis and repair. Cells possess an innate ability to share mitochondria with neighboring cells *via* various mechanisms, including tunneling nanotubes, extracellular vesicles, and other forms of cell-to-cell contact [90,91]. This transfer serves as a vital rescue mechanism, allowing healthy cells to support distressed neighbors by replenishing their mitochondrial pool. Research has demonstrated the critical role of this process in the bone marrow microenvironment. For instance, in the aged bone marrow niche, a phenomenon characterized by systemic senescence and reduced regenerative capacity, rejuvenating senile macrophages *via* a cerium-based nanosystem activated the SIRT1-PGC-1α axis. This activation did not merely improve the health of the macrophages themselves; it crucially stimulated de novomitochondrial biogenesis within these immune cells. Subsequently, these newly generated healthy mitochondria were efficiently transferred to neighboring aged BMSCs, effectively restoring a more youthful osteogenic phenotype and combating the functional decline associated with aging [75]. Furthermore, bioengineering approaches are being developed to actively promote this beneficial process. Engineered biomaterials can be designed to create a microenvironment that fosters and directs mitochondrial donation. A prime example is an immune-responsive hierarchical hydrogel that was specifically designed to adapt to the bone regeneration environment. This sophisticated material facilitated continuous, unidirectional mitochondrial transfer from macrophages to BMSCs, thereby providing essential and sustained bioenergetic support to the stem cells during the demanding process of bone formation [92]. This cell-mediated approach leverages the body’s own cellular ‘factories’ to produce and distribute healthy mitochondria, representing a highly sophisticated and physiologically integrated form of therapy. However, major challenges for clinical translation remain. These include the low baseline efficiency of natural transfer, the difficulty in precisely controlling the quantity and specificity of transferred mitochondria *in vivo*, and the scalability of bioengineering solutions to human-scale defects.

#### 3.4.2. Direct mitochondrial transplantation

Moving beyond leveraging endogenous cellular processes, direct mitochondrial transplantation involves the external isolation and subsequent transplantation of intact, functional mitochondria from a healthy source into damaged or dysfunctional recipient cells or tissues. This method is akin to an ‘organelle transplant’. The protocol typically involves isolating mitochondria from healthy, metabolically active donor cells, such as autologous or allogeneic BMSCs, and then delivering them to the target site. Evidence for its efficacy is robust in *ex vivo* settings. For example, mitochondria isolated from healthy donor BMSCs can be transplanted *in vitro* into recipient BMSCs that have compromised function [93]. This procedure has been shown to significantly boost the recipient cells’ OXPHOS activity, ATP production, and ultimately, their osteogenic capacity. Importantly, this enhancement was not merely a correlative finding; it translated to improved bone formation in a rat critical-size bone defect model. The causal link to metabolism was conclusively demonstrated when the pro-osteogenic effect was abrogated by the administration of oligomycin, a specific OXPHOS inhibitor, confirming that the benefits were directly dependent on the restoration of energy metabolism [94–96]. To translate this promising approach to clinical *in vivo* applications, innovative delivery systems are required to protect the fragile organelles during systemic circulation and ensure targeted delivery. One such advanced system is the Mitochondria-loaded Erythrocyte (MiLE) system. MiLEs act as sophisticated biological carriers, utilizing the patient’s own red blood cells to encapsulate the isolated mitochondria. This encapsulation protects the mitochondria during storage and delivery, shields them from immune recognition, and remarkably, allows for the co-delivery of oxygen bound to hemoglobin within the same erythrocyte. In an inflammatory bone destruction model, the administration of MiLEs produced a multifaceted therapeutic outcome: it not only provided metabolic rescue but also inhibited osteoclastogenesis and promoted polarization of macrophages towards the anti-inflammatory M2 phenotype [97]. This demonstrates that direct mitochondrial transplantation can exert potent immunomodulatory effects alongside its primary metabolic rescue function, making it a highly versatile strategy for complex inflammatory bone disorders. Nevertheless, significant technical hurdles persist. The isolation process must maintain mitochondrial membrane integrity and functionality. After transplantation, ensuring the long-term survival, proper integration, and avoidance of immune rejection of allogeneic mitochondria are critical, unresolved issues for clinical application.

#### 3.4.3. Exosome-mediated delivery of key components

An alternative to the logistical challenges of transplanting entire organelles is the use of extracellular vesicles, particularly exosomes, as natural nanocarriers to deliver specific, critical mitochondrial components. This strategy represents a more targeted, molecular-level ‘reconstitution’ rather than whole-scale replacement. Exosomes are small vesicles secreted by cells that carry a cargo of proteins, lipids, and nucleic acids, and play a key role in intercellular communication. They can be engineered or harnessed from specific cell types to carry therapeutic payloads. A compelling example of this approach utilizes exosomes derived from stem cell aggregates of exfoliated deciduous teeth. These exosomes were found to be naturally enriched in the mRNA encoding for Mitochondrial Transcription Factor A (TFAM). TFAM is a nuclear-encoded protein that is absolutely essential for mitochondrial DNA transcription and replication, acting as a master regulator of the mitochondrial genome. Upon delivery to recipient cells, such as dental pulp stem cells (DPSCs), this exogenous TFAM mRNA leads to a significant increase in TFAM protein expression within the recipient’s mitochondria. This upregulation, in turn, enhances metabolic pathways like glutamate metabolism and, most critically, boosts OXPHOS activity. The net result is a significant improvement in the bone regeneration capacity of the recipient stem cells, achieved by activating mitochondrial aerobic metabolism from within the existing network [98]. This method bypasses the need to handle fragile whole organelles and offers a highly targeted, gene therapy-like approach to rebuild mitochondrial function by supplementing its fundamental regulatory machinery. A key challenge lies in the precise engineering and quality control of exosomes to ensure consistent therapeutic cargo loading, efficient cellular uptake, and reproducible biological effects across different patient-derived cells and microenvironments.

#### 3.4.4. Modulating endogenous transfer pathways

**Figure 6.**
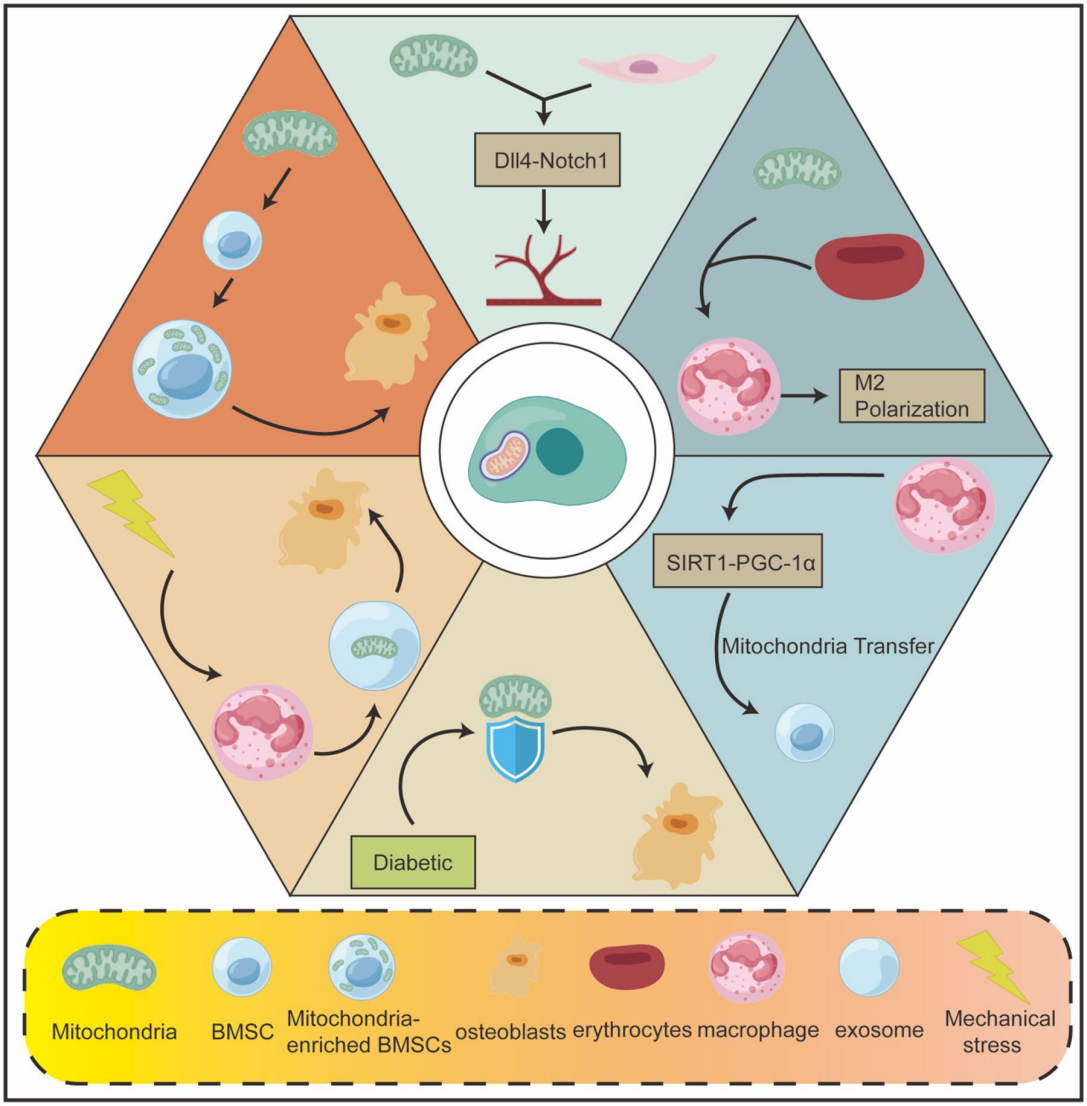
Therapeutic strategies targeting mitochondria.

Instead of directly administering mitochondria or their components, a highly promising alternative strategy involves pharmacologically targeting the endogenous cellular signaling pathways that naturally govern mitochondrial transfer [99]. The rationale is that enhancing the body’s own inherent mitochondrial ‘sharing’ mechanisms could be a more subtle and potentially safer therapeutic approach [100]. Achieving this requires a deep understanding of the molecular signals that control this process. A significant discovery in this area was the identification of the kinase RIPK4 as a key regulator of mitochondrial dynamics and transfer within the bone marrow niche. It was found that RIPK4 physically interacts with and promotes the degradation of the mitochondrial fusion protein MFN2. By negatively regulating MFN2, RIPK4 disrupts the normal balance between mitochondrial fission and fusion. Furthermore, this RIPK4-MFN2 interaction was shown to be a critical regulator of mitochondrial transfer from osteolineage cells to hematopoietic cells, thereby influencing bone marrow myeloid hematopoiesis [101]. This discovery unveils a novel signaling axis (RIPK4-MFN2) that could be targeted therapeutically; for instance, inhibiting RIPK4 activity could stabilize MFN2, promote a more fused mitochondrial network, and potentially enhance the efficiency of natural or therapy-driven mitochondrial transfer between cells. This approach highlights the future potential of modulating endogenous transfer pathways to improve the efficacy of other mitochondrial replacement strategies or to stimulate the body’s own reparative capabilities as a standalone treatment. The primary challenge for this strategy is achieving sufficient specificity to modulate mitochondrial transfer within the complex bone repair niche without affecting essential mitochondrial functions in other cell types or triggering off-target effects, which necessitates the development of highly targeted delivery systems for such modulators (Figure 6).

This figure systematically illustrates mitochondrial-targeted bone repair therapeutic strategies, including promoting angiogenesis through Dll4-Notch1 signaling, regulating macrophage polarization toward the M2 phenotype, activating the SIRT1-PGC-1α pathway to enhance mitochondrial biogenesis, and utilizing mitochondrial transfer and mitochondria-enriched BMSCs to directly improve cell function, thereby synergistically promoting osteogenic differentiation and tissue regeneration under conditions such as diabetes or mechanical stress.

## 4. Challenges and future outlook

Compared to conventional bone repair strategies like stem cell transplantation or growth factor delivery, which primarily supply cellular components or extracellular signals, mitochondrial-targeted therapy represents a more fundamental approach aimed at restoring the intrinsic functional capacity of resident and recruited cells. Mitochondrial-targeted therapeutic strategies represent a paradigm shift in bone regenerative medicine, demonstrating remarkable potential in preclinical models. However, the translation of these promising approaches from fundamental research to widespread clinical application is fraught with significant challenges that necessitate a multidisciplinary effort to overcome. The primary hurdle remains achieving efficient and cell-type-specific delivery within the complex bone repair microenvironment. Current delivery systems often lack the precision to accumulate effectively at the defect site and subsequently target the mitochondrial compartment of specific cell populations, such as MSCs or osteoblasts, while avoiding off-target effects on osteoclasts or immune cells [90]. Secondly, the long-term safety profile and potential systemic toxicities of chronic mitochondrial modulation demand urgent and thorough investigation [102]. As central regulators of metabolism and cell death, mitochondrial interventions could unpredictably interfere with systemic energy homeostasis or trigger adverse immune responses. Furthermore, the molecular mechanisms driving mitochondrial dysfunction in various bone pathologies are heterogeneous and not fully elucidated, leading to a scarcity of sufficiently precise therapeutic targets and hindering the development of personalized treatment regimens.

Looking ahead, breakthroughs in this field will hinge on interdisciplinary convergence and technological innovation across several key fronts. First, the development of next-generation, intelligent delivery systems is paramount. This includes leveraging bone-homing peptides for tissue-specific targeting and designing sophisticated microenvironment-responsive materials that release their cargo in response to pathological cues such as abnormal pH, elevated ROS, or specific enzymes present in the repair niche [103]. These strategies will significantly enhance targeting precision and therapeutic efficacy. Second, future therapies must move beyond single-target approaches towards multi-target synergistic strategies. A holistic ‘metabolism-ROS-immunity’ triad approach is warranted. For instance, a combinatory strategy might simultaneously enhance energy production, scavenge excess ROS, and modulate the inflammatory response, thereby comprehensively restoring mitochondrial and immune homeostasis. A critical aspect of this approach, particularly for redox modulation, is defining the precise, cell type-specific ROS thresholds that separate beneficial signaling from oxidative damage in the bone regenerative niche. This remains a key challenge, as current studies often lack the resolution to define exact concentration ranges for different cell types at various repair stages. Future progress will depend on developing more sensitive in situdetection technologies and sophisticated in vitromodels to map these dynamic thresholds. Deeper integration with immunology is critical, specifically exploring the role of mitochondrial function in immune cells within the skeletal system. Understanding how mitochondrial metabolism in macrophages and T cells dictates their fate and function—and consequently influences the ‘osteo-immune’ environment—will unveil novel targets for immunomodulatory bone therapies.

The introduction of emerging technologies will be a major catalyst. Advanced models like bone-specific organoids and ‘bone-on-a-chip’ microphysiological systems provide unprecedented opportunities to study mitochondrial dynamics and screen drug candidates in a human-relevant, pathomimetic microenvironment [104,105]. Furthermore, single-cell multi-omics technologies can unravel the previously obscured mitochondrial heterogeneity between different cell types in the bone marrow, identifying distinct functional states and vulnerabilities. Artificial intelligence and machine learning are poised to revolutionize the field by accelerating the design and virtual screening of novel mitochondrial-targeting drugs and smart biomaterials, predicting their efficacy and potential toxicities before costly synthesis.

The clinical translation pathway should be strategically planned. The short-term focus must be on rigorous safety and biodistribution profiling of lead candidates in advanced human-cell-based models and large animals. The mid-term goal should involve conducting well-designed proof-of-concept studies in relevant animal models of bone disease, followed by early-phase clinical trials to establish safety and preliminary efficacy in specific patient populations. The long-term vision is the realization of personalized mitochondrial medicine for skeletal disorders. This will involve defining mitochondrial function biomarkers for patient stratification and developing customizable biomaterial platforms that can be tailored to an individual’s specific mitochondrial deficit profile, ultimately achieving precise and effective bone regeneration.

## 5. Conclusion

Mitochondrial-targeted strategies for bone repair mark a transformative advancement in regenerative medicine by shifting the therapeutic focus to the core of cellular energetics. This review has systematically aligned specific pathological mechanisms—bioenergetic deficits, oxidative stress, dynamical instability, and irreparable damage—with their corresponding intervention strategies: metabolic enhancement, antioxidant and redox regulation, dynamics and quality control remodeling, and functional replacement. This review provide a rational framework for developing treatments.

The future of this field lies in embracing a cross-disciplinary approach to overcome existing challenges in delivery precision, long-term safety, and patient-specific responses. The convergence of material science, immunology, and data science will be critical. The ultimate goal is to transition from a one-size-fits-all approach to personalized mitochondrial medicine. The clinical translation pathway involves short-term safety validation in advanced models, mid-term proof-of-concept clinical studies for specific bone disorders, and the long-term development of combinatory, multi-targeted therapies tailored to an individual’s mitochondrial functional profile. By systematically addressing mitochondrial dysfunction at its root, these strategies hold the promise of ushering in a new era of precise and effective treatments for challenging skeletal conditions.

## Data Availability

There is no data associated with this research.
